# Inhibitory effects of glutathione peroxidase on microbial spoilage of crayfish (*Procambarus clarkii*) during refrigerated storage

**DOI:** 10.1016/j.fochx.2024.101388

**Published:** 2024-04-13

**Authors:** Lerong Qin, Han Li, Ying Zhang, Jiwang Chen, Haibin Wang, E Liao

**Affiliations:** a College of Food Science and Engineering, Wuhan Polytechnic University, Wuhan 430023, China; b Hubei Key Laboratory for Processing and Transformation of Agricultural Products (Wuhan Polytechnic University), Wuhan 430023, China; c National Research & Development Branch Center for Crayfish Processing (Qianjiang), Qianjiang 433100, China; d National Research & Development Center for Se-rich Agricultural Products Processing Technology, Wuhan, 430023, China

**Keywords:** Crayfish, Glutathione peroxidase, 16S rRNA, Bacterial composition, Microbial spoilage, Trichloroacetic acid (PubChem CID: 6421), Trimethylamine (PubChem CID: 1146), Sodium hydroxide (NaOH) (PubChem CID: 14798), Boric acid (PubChem CID: 7628), Hydrochloric acid (PubChem CID: 313), Formaldehyde (PubChem CID: 712), Eoxynucleoside triphosphates (dNTPs) (PubChem CID: 155489856), Agarose (PubChem CID: 11966311)

## Abstract

The variety of enzyme-based biological preservatives is limited. This study evaluated the effects of glutathione peroxidase (GSH-Px) on the quality of crayfish during refrigerated storage by measuring the pH, total volatile basic nitrogen, trimethylamine, and microbial contamination in crayfish muscle simulation system. The results revealed that 0.3% GSH-Px (CK3) not only suppressed the degradation of nitrogenous substances but also decreased the contamination levels of total viable, Enterobacteriaceae, and *Pseudomonas* counts (*P* < 0.05). Furthermore, the populations of *Lactococcus*, *Aeromonas*, and *Massilia* differed in the CK3 group compared to the other groups (*P* < 0.05) at the end of the storage (day 15). Moreover, the principal coordinate analysis showed that the colony composition of CK3 stored for 15 days was similar to that of the control group stored for 10 days. Therefore, GSH-Px exhibits antibacterial activity against Gram-negative bacteria and has good application potential in freshwater aquatic product preservation.

## Highlights

• The glutathione peroxidase (GSH-Px) exhibited antibacterial activity for crayfish during refrigerated storage.
• The relative abundances of *Shewanella* and *Hafnia-Obesumbacterium* were significantly inhibited after GSH-Px treatment.
• The differences of colony structure among groups have analyzed by LDA discrimination.
• The GSH-Px has good application potential in crayfish product refrigerated preservation.

## 1 Introduction

Crayfish (*Procambarus clarkii*) is one of the most popular freshwater aquatic food in China, and the output has reached 2.63 million tons in 2021 (Liao et al., 2023). Due to the high moisture content and abundant protein, crayfish is susceptible to microbial contamination during storage and transportation, and various preservatives are usually applied to suppress such undesirable phenomena (Ge et al., 2020; Zhang, Mao, Yao, & Aubourg, 2020; Zhang, Zhao, Chen, Zhang, & Wei, 2019). In the food preservation industry, there is a growing trend towards the development of natural, safe, and non-toxic methods (Peng et al., 2022), particularly focusing on enzyme-based biological preservatives due to their low dosage, high efficacy, and good biocompatibility (Mei, Ma, & Xie, 2019; Wang, Hu, Gao, & Wang, 2017; Yuan et al., 2020). Among them, glucose oxidase P5 showed a good antibacterial effect on common aquatic product pathogens of *Listeria monocytogenes* and *Vibrio parahaemolyticus* (Yuan et al., 2020). Lysozyme mainly acts on Gram-positive bacteria (Mei et al., 2019), and Wang et al. (2017) used collagen and lysozyme as edible coatings for fresh salmon to significantly improve the quality of salmon fillets at 4 ± 1 °C for 15 days. Additionally, lactoperoxidase generally exhibits antibacterial activity against Gram-negative bacteria (Mei et al., 2019), and Shokri and Ehsani (2017) applied the lactoperoxidase system in the storage of pike-perch fillets at 4 °C, extending their shelf lives to day 16. However, the types of enzymatic preservatives are still limited.

Selenium is an essential trace element for higher organisms (Wan, Zhang, & Adhikari, 2018), and has been proven to have important biological functions, including antioxidant, anti-cancer, detoxification, and immunity enhancement (Zhou et al., 2018). Researchers have so far conducted antibacterial studies on various forms of selenium, focusing on inorganic selenium (selenite) (Cermelli et al., 2002) and selenium nanoparticles (SeNPs) (zero-valent elemental selenium) (Han et al., 2021). In contrast, biosynthetic SeNPs derived from plants, bacteria and fungi cultured in selenium-fortified substrates have a better safety profile (Han et al., 2021). The synthesized SeNPs by dos Santos Souza et al. (2022) from extracts of three plants, onion (*Allium cepa*), acerola (*Malpighia emarginata*) and boldo (*Gymnanthemum amygdalinum*) inhibit Gram-positive bacteria, while SeNPs biosynthesized in cells of *Lactobacillus pentosus* ADEB had strong antagonistic activity against the Gram-negative bacteria *Salmonella arizonae* (Adebayo-Tayo, Yusuf, & Alao, 2021). Meanwhile, due to its favorable biological activity and safety profile, organic selenium has also attracted much research attention recently on its bacteriostatic ability. For instance, Methicillin-resistant *Staphylococcus aureus* and *Escherichia coli* could be suppressed by β-selenoamines and diselenide compounds (Stefanello et al., 2020). As for naturally sourced selenoproteins from animals and plants, a large number of studies have focused on their physiological functions in organisms, such as the effects on animal growth performance and immune function (Zhang et al., 2020), effects on oxidative stress, inflammation and other diseases (Li et al., 2020), and mitigation effects on arsenic and cadmium biotoxicity (Zwolak, 2020). Besides, partial studies aim to reveal the composition, structure and antioxidant activity of selenoproteins from different sources (Wu et al., 2020; Zhou et al., 2018). However, there is limited research on the capacity of selenoproteins to regulate microorganisms.

Our previous proteomic analysis revealed a significant upregulation of glutathione peroxidase (GSH-Px) abundance in crayfish muscle during superchilling storage (unpublished data). GSH-Px (EC 1.11.1.9) is a typical selenoprotein found in nearly all organisms. It functions as a reducing agent in various biochemical reactions, including the reduction of cell membrane lipids, thereby playing a crucial role in protecting organisms from oxidative damage (Brigelius-Flohé & Flohé, 2020). Considering the properties of the selenium-containing compounds mentioned earlier, it is speculated that the up-regulation of GSH-Px in superchilled crayfish could positively influence the storage characteristics of crayfish.

To explore the effect of GSH-Px on the storage quality of crayfish in comprehensively, varying concentrations (0.1%, 0.3%) of GSH-Px were exogenously added in crayfish muscle to assess the effects on the degradation of nitrogenous substances and the proliferation of microorganisms. Additionally, high-throughput sequencing of 16S rRNA was conducted to evaluate the effect of GSH-Px on the microbial community dynamics during the storage of crayfish muscle.

## 2 Materials and methods

### 2.1 GSH-Px solution and crayfish muscle simulation system (CMSS) preparations

For activation of GSH-Px (130 U/mg; Sigma-Aldrich, Darmstadt, Germany), the enzyme of 1.0 mg was dissolved in the sterile aqueous solution of 10.0 mL. Then, the enzyme solutions (2.5 mL and 7.5 mL) were added to sterile aqueous solutions to final concentrations (0.1%; 0.3%), and their theoretical enzyme activities were 32.5 U and 97.5 U, respectively.

The purchased 10 kg alive crayfish (weight: 19.8–25.5 g; length: 9.5–11.5 cm) from Wushang Supermarket in Wuhan, China (Jun 2021) were placed in a foam box containing crushed ice and transported to the laboratory within 30 min. Subsequently, the crayfish were sonicated in ultrasonic cleaner with tap water for 30 min at 25 kHz, and then kept in ice for cold shock until use (no >3 h). The crayfish were peeled and immediately put into crushed ice. After all of the crayfish had been processed, the rinse of the surface was performed via pre-chilled sterile water. After draining on a sterile stainless-steel mesh for 10 min, the crayfish were randomly divided into three groups (about 200 g crayfish in each group), which were homogenized by a high-speed disperser (Scientz, Ningbo, China) at 8000 r/min for 30 s in a solution (2:1, *w*/*v*) containing 0%, 0.1%, and 0.3% GSH-Px, respectively. All above operations were carried out on a sterile operating table. The resulting CMSS was named CT, CK1, and CK3, respectively. The three groups of crayfish homogenates were individually packed in sterile high-density polyethylene sealed boxes (thickness: 2 mm) and stored in the refrigerator at 4 °C. On days 0, 3, 6, 10, and 15 during storage, samples were drawn from the three groups for physicochemical analysis, total DNA extraction, and PCR amplification.

### 2.2 Determination of pH

The detection of pH value was performed in the homogenate, which was obtained after the high-speed dispersion of crayfish muscle (5 g) and deionized water (50 mL) for 1 min at 8000 r/min. This method was from López-Caballero, Martínez-Alvarez, Gómez-Guillén, and Montero (2007) with minor modifications, and a digital pH-meter (FE28-Standard, Metler-Toledo, Shanghai, China) was used to determine the pH value.

### 2.3 Determination of total volatile basic nitrogen (TVB-N) and trimethylamine (TMA—N) contents

The ascertainment of TVB-N and TMA contents was carried out by the method of Qin et al. (2022). The homogenate, a mixture of minced muscles(10 *g*)and 20 g/L trichloroacetic acid (50 mL), was made at a speed of 946 ×g for 1 min by a high-speed disperser (Scientz, Ningbo, China). Before the 10 min of standing still, the vortex was conducted for 1 min at a speed of 3000 r/min by the vortex shaker (Xinbao, Jiangsu, China). This process was repeated twice. Filtrate (10 mL), 10% (*w*/*v*) NaOH solution (5 mL), and deionized water (10 mL) were used for 5 min of distillation in a Kjeldahl-type distillation tube, and the distillate was imported into 20 g/L aqueous boric acid solution (10 mL) and titrated by 0.01 M hydrochloric acid standard titrant. It was worth noting that 35% (*v*/v) formaldehyde (20 mL) was poured before NaOH solution in the admeasurement method of TMA content. The final results were expressed as mg/100 g crayfish muscle.

### 2.4 Microbiological analysis

Microbial contamination was evaluated by total viable counts (TVC), Enterobacteriaceae counts (EBC), and *Pseudomonas* counts (PDC). The ground sample (25 g) was aseptically diluted with 0.9% NaCl (225 mL) and mixed for 1 min via Vortex mixer (Servicebio, Wuhan, China), followed by the preparation of a series of 10-fold dilutions. One milliliter of dilution was inoculated in corresponding growth media: TVC on plate count agar at 30 °C statically for 48 h; EBC on violet red bile agar at 37 °C statically for 24 h using the double-layer technique (Balti et al., 2020); PDC on *Pseudomonas* Cetrimide-Fucidin-Cephaloridine Selective Agar at 25 °C statically for 48 h. After the raw data analyzed statistically, the mean values of colony-forming units per gram were converted to log_10_ CFU/g to express the colonies number.

### 2.5 DNA extraction and polymerase chain reaction (PCR) amplification

Total genomic DNA was extracted from 13 samples (Fresh, CT_3d, CK1_3d, CK3_3d, CT_6d, CK1_6d, CK3_6d, CT_10d, CK1_10d, CK3_10d, CT_15d, CK1_15d, CK3_15d) using the TGuide S96 Magnetic Universal DNA Kit (TIANGEN BIOTECH CO., Beijing, China). A synergy HTX microplate reader (Gene Company Limited, Hong Kong, China) was used to detect the concentration of the extracted nucleic acid to evaluate the quality and quantity of the extracted DNA.

The PCR amplification was performed according to the method of Zang et al. (2018), which aimed at the V3-V4 region of the bacterial 16S rRNA gene and relied on the universal primers 338F (5’-ACTCCTACGGGAGGCAGCA-3′) and 806R (5’-GGACTACHVGGGTWTCTAAT-3′). The first thermal cycle took place in a total volume of 10 μL system (containing 5 μL buffer, 0.2 μL KOD FX Neo PCR enzyme, 2 μL dNTPs, 3 μM of each primer, and 50 ng genomic DNA) with an initial denaturation at 95 °C for 5 min, followed by 15 cycles at 95 °C for 30 s, 50 °C for 30 s and 72 °C for 40 s and a final extension at 72 °C for 7 min.

Subsequently, 5 μL of PCR product purified by VAHTS™ DNA Clean Beads magnetic beads were added to a 40 μL reaction system along with 10 μL of 2 × Q5 HF MM and 2.5 μL of each primer for the second round of PCR. The program of thermal cycling was a denaturation at 98 °C for 30 s first, followed by 10 cycles of 98 °C for 10 s, 65 °C for 30 s and 72 °C for 30 s, and finally a denaturation at 72 °C for 5 min. The 1.8% agarose gel electrophoresis method was carried out to purify and recover the amplified products.

### 2.6 Sequencing of the 16S rDNA and data preprocessing

The 16S rRNA gene amplicons were sequenced on the Illumina Novaseq 6000 sequencing platform by Biotree Biomedical Technology Co., (Shanghai, China). For data preprocessing, the filtration of the sequenced raw reads was performed by employing Trimmomatic v0.33 software and then the identification and removal of primer sequences were actualized by cutadapt 1.9.1 software to obtain the clean reads. To secure the final effective reads, clean reads were assembled by Usearch v10 software and its chimeras were identified using the UCHIME algorithm (Edgar, Haas, Clemente, Quince, & Knight, 2011).

### 2.7 Bioinformatics analysis

Sequences were bioinformatically analyzed using the Quantitative Analysis of Microbial Ecology (QIIME) package (Caporaso et al., 2010). Reads were clustered at a similarity level of 97.0% using Usearch software (Edgar, 2013) to obtain operational taxonomic units (OTUs). Taking SILVA as the reference database, the feature sequence taxonomically was annotated using the Naive Bayes classifier. The alpha diversity (OTUs, Ace, Chao1, Simpson, Shannon, and Goods coverage indexes) was determined by Mothur v.1.30.1 with a 97% similarity. The beta diversity, including hierarchical clustering (by unweighted pair-group method with arithmetic means), principal coordinates analysis (PCoA), and heatmap, was ascertained using QIIME software. Further, multi-level species difference discriminant analysis (linear discriminant analysis, LDA) was performed by LEfSe software and Kyoto Encyclopedia of Genes and Genomes (KEGG) function prediction (http://www.genome.jp/kegg/) was defined on the 16S amplicon sequencing results by PICRUSt2 software.

### 2.8 Data analysis

All analyses were conducted in triplicate, and the data were expressed as the mean ± standard deviation from multiple groups of independent experiments. Differences among mean values were analyzed with one-way analysis of variance (ANOVA) followed by Duncan's post hoc test using the software SPSS Version 25 (SPSS Inc., Chicago, IL, USA), and the significant deviation was defined as a significance level of *P* < 0.05.

## 3 Results and discussion

### 3.1 Effects of GSH-Px on pH in CMSS

**Fig. 1:**
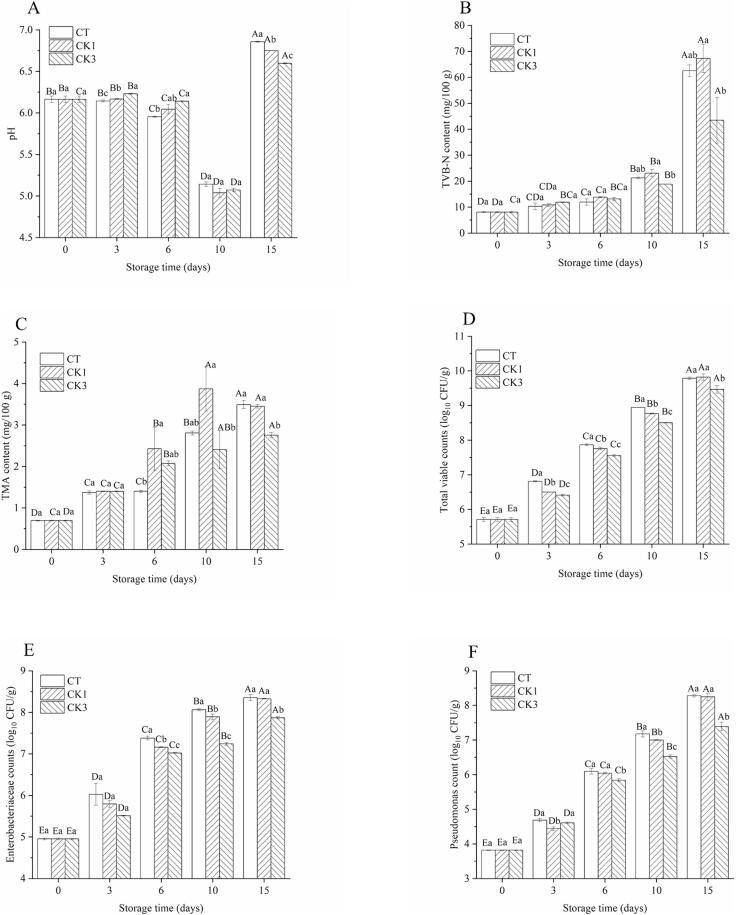
The pH (A), TVB-N content (B), TMA content (C), total viable bacteria count (D), Enterobacteriaceae counts (E), and *Pseudomonas* count (F) in samples. CT: samples without glutathione peroxidase; CK1: samples with 0.1% glutathione peroxidase; CK3: samples with 0.3% glutathione peroxidase. The different uppercase letters within the same treatment indicate significant differences (*P* < 0.05). The different lowercase letters within the same storage time indicate significant differences (*P* < 0.05).

The effects of varying concentrations of GSH-Px on the pH in CMSS were investigated, and the results are depicted in Fig. 1(A). The initial pH value of CMSS (6.16) was lower than that of fresh crayfish (6.44) reported by Qin et al. (2022). This disparity may be attributed to the acceleration of glycolysis in CMSS following homogenization treatment accompanied by the high temperature, leading to the tremendous accumulation of acidic substances or hydrogen ions. The pH in each group showed a trend of increasing after decreasing on the whole. An increase in pH was observed with the rise in GSH-Px concentration during the initial 6 days (*P* < 0.05). This phenomenon can be attributed to the property of GSH-Px, which converts slightly acidic substrates (such as H_2_O_2_) into neutral substances (such as H_2_O) (Brigelius-Flohé & Flohé, 2020). However, there were no significant differences (*P* > 0.05) observed among the three groups on day 10. After 15 d storage, the pH values increased to 6.86, 6.75 and 6.60 in CT, CK1 and CK3 groups, respectively, with significant differences (*P* < 0.05) observed among them. These observations suggested that GSH-Px may regulate the rates of glucose and nitrogen metabolism in crayfish muscle, as the pH value reflects a balance between the acidic substances from sugar metabolism and the alkaline substances from the degradation of nitrogenous substances (Ge et al., 2020).

### 3.2 Effects of GSH-Px on TVB-N and TMA in CMSS

To investigate whether the GSH-Px could regulate the rate of nitrogen metabolis, changes in TVB-N and TMA contents were monitored in CMSS during storage. As depicted in Fig. 1(B, C), the initial TVB-N and TMA values in CMSS were 8.10 and 0.69 mg/100 g, respectively, on day 0, and then their levels increased continuously during storage. For TVB-N, there was no significant difference (*P* > 0.05) observed between the CK1 and CT groups within 15 days. However, the CK3 group (18.85 mg/100 g) exhibited a lower content compared to the CK1 group (23.07 mg/100 g) after 10 days storage (*P* < 0.05). It could be proven that 0.3% of GSH-Px had a remarkable inhibitory effect on the degradation of nitrogenous substances in crayfish muscle (≥ 10 d). TMA, an important volatile amine, did not show significantly differentiated until the end of storage in the CK3 (2.75 mg/100 g) and CT (3.49 mg/100 g) groups. However, accumulation in the CK1 group exceeded that of the CT group on day 6 (*P* < 0.05). Therefore, the effects of GSH-Px on TVB-N and TMA were dependent on its concentration. In summary, the role of GSH-Px in the later stages of storage may be attributed to several factors: 1) significant degradation of nitrogen-containing substances after 10 days of storage, leading to a rapid rise in CMSS pH, making it more conducive for GSH-Px activity (data not shown); 2) when active oxygen or peroxide levels in CMSS exceeded a certain threshold, dicarbonyl compounds such as malondialdehyde produced by lipid rancidity could modify GSH-Px, reducing its Michaelis-Menten constant (*Km*) to augment the affinity of the enzyme for its substrate, thereby increasing enzyme activity (Lankin, Shumaev, Tikhaze, & Kurganov, 2017); 3) variations in enzyme concentration could affect the availability of the substrate, thereby impacting reaction kinetics.

### 3.3 Effects of GSH-Px on the microbiological characteristics in CMSS

The degradation of nitrogenous substances, including pyrimidines, purines, proteins, and others, is closely related to the activity of microorganisms (Wang, Chen, Zhang, Wang, & Shi, 2020). To explore the preservation mechanism of GSH-Px, the changes of microorganisms in CMSS incorporated with different concentrations of GSH-Px during storage were studied. The assay results of TVC, EBC, and PDC were summarized in Fig. 1 (D—F). It was observed that GSH-Px was able to inhibit microbial growth, although to varying degrees.

TVC in all groups increased during storage (Fig. 1D). However, compared to CT, exogenous GSH-Px significantly (*P* < 0.05) decreased the TVC in CMSS. The TVC difference between CK1 and CT groups disappeared (*P* > 0.05) up till day 15. Meanwhile, this difference persisted in CK3 and other groups (*P* < 0.05). Hence, enzyme concentration is an important factor affecting its duration of action. According to the report by Gram and Dalgaard (2002), TVB-N including TMA originated from microbial metabolism, of which Enterobacteriaceae-like organisms were one of the major contributors. The accumulation of Enterobacteriaceae in CK3 (7.87 log_10_ CFU/g) was lower (*P* < 0.05) than that in CK1 (8.33 log_10_ CFU/g) and CT (8.36 log_10_ CFU/g) after storage (Fig. 1E). Notably, the EBC level on day 6 in the CK1 group (7.16 log_10_ CFU/g) was comparable to that in the CK3 group (7.24 log_10_ CFU/g) on day 10, and a similar trend was observed between the CK1 group on day 10 and the CK3 group on day 15 (7.89 and 7.87 log_10_ CFU/g). Therefore, 0.3% of GSH-Px established a more potent bacteriostatic effect than 0.1% of GSH-Px. Psychrotrophic bacteria are the most active microorganisms that alter the quality of refrigerated aquatic products (Balti et al., 2020), and especially *Pseudomonads* spp. was considered as a representative microorganism of shrimp spoilage (Na, Kim, Jang, Park, & Oh, 2018). As with other bacteria, PDC was also affected by the concentration of GSH-Px. The primary PDC level (3.82 log_10_ CFU/g) was raised to 8.78, 8.25, and 7.30 log_10_ CFU/g in CT, CK1, and CK3 groups in the final CMSS, respectively (Fig. 1F), manifesting that GSH-Px was in a position to drastically reduce *Pseudomonas* growth in CMSS (≥ 6 d). Overall, the application of GSH-Px as a preservative presents a promising alternative to extend the shelf life of crayfish by reducing the growth rate of various bacteria.

### 3.4 Bacterial community Richness and diversity in CMSS

Further analysis of the bacterial community affected by GSH-Px was conducted using high-throughput sequencing. As shown in Table 1, a total of 1,007,650 pairs of sequences were obtained from 39 samples sequenced by Illumina Novaseq 6000. After further double-ended read splicing and screening, the number of clean tags in all samples exceeded 60,000 (the effective percentage ≥ 90%), and the average length ranged from 423.33 bp to 429.00 bp. Thus, the clean tags of the samples were adequate for subsequent analysis of microbial richness and diversity.

**Table 1:** Sequence abundance and microbial diversity in samples at 0, 3, 6, 10, and 15 days of refrigerated storage. CT: samples without glutathione peroxidase; CK1: samples with 0.1% glutathione peroxidase; CK3: samples with 0.3% glutathione peroxidase.

Samples	Raw read	Clean tags	Number of OTUs	Chao 1 index	Ace index	Shannon index	Simpson index	Good's coverage
Fresh	74,427	74,189	1137.67	1378.52	1394.40	4.87	0.06	0.99771
CT_3d	77,783	77,482	963.33	1184.20	1084.16	3.96	0.07	0.99785
CK1_3d	68,834	68,563	926.33	1133.00	1050.07	3.89	0.08	0.99749
CK3_3d	73,946	73,629	1024.67	1217.26	1128.09	4.14	0.05	0.99768
CT_6d	78,491	78,189	744.67	992.63	961.65	2.99	0.09	0.99671
CK1_6d	80,047	79,742	766.67	933.86	912.78	2.99	0.10	0.99737
CK3_6d	79,927	79,616	966.00	1145.16	1107.22	3.07	0.11	0.99707
CT_10d	80,017	79,754	650.67	856.31	875.95	2.70	0.15	0.99701
CK1_10d	78,400	78,150	562.67	757.70	801.27	2.65	0.14	0.99711
CK3_10d	79,827	79,574	492.00	691.00	679.21	2.42	0.18	0.99771
CT_15d	77,303	77,042	543.67	741.06	887.62	2.73	0.15	0.99721
CK1_15d	79,557	79,287	645.00	809.85	835.07	2.70	0.16	0.99728
CK3_15d	79,091	78,866	365.00	498.67	578.07	2.54	0.17	0.99826

The clean tags with sufficient similarity were assigned to OTUs after clustering/denoising, and then statistical analysis of the richness and diversity of microorganisms in crayfish samples was conducted based on OTUs information at the 97% similarity level (Table 1). OTUs, Chao1 index (estimated OTUs in the sample), and Ace index (estimated OTUs in the community) typically reflect the richness of the microbial community. The highest values were observed in the fresh samples (1137.67, 1394.40, 1378.52) for all these indexes. With the prolongation of refrigeration, the gradual diminishment of microbial richness in CMSS was proved by the overall decreasing trend of OTUs, Chao1, and Ace index. These indexes in CK3 group were higher than those in groups of CT and CK1 within 6 days, while they turned to a minimum after 10 days. It is evident that 0.3% of GSH-Px had a substantial impact on the microbiota structure of CMSS during refrigeration, particularly in reducing microbial richness in the later refrigeration period (*P* < 0.05).

As estimators of sample diversity, the Shannon and Simpson indexes represent the approximate number of species and the uniformity of their distribution in the sample. The higher Shannon and the lower Simpson index stand for higher community diversity. Bacterial diversity in each group tended to decline as storage progressed. After 0.3% of GSH-Px treatment, the microbial diversity of CMSS decreased slowly within 10 days, and then rapidly decreased after 10 days, which was consistent with the trend of microbial richness. This might be attributed to the strong inhibitory effect of high acidity conditions on the exogenous reproduction of microorganisms from day 10.

In addition, the coverage index in all samples was >99%, indicating that enough bacteria were found and the sequencing results could reflect the actual situation. At the same time, the storage periods could be divided into early (0 d, 3 d), middle (6 d), and later (10 d, 15 d) stages according to the characteristics of OTUs. There were significant changes among the regions.

### 3.5 Bacterial community composition in CMSS

**Fig. 2:**
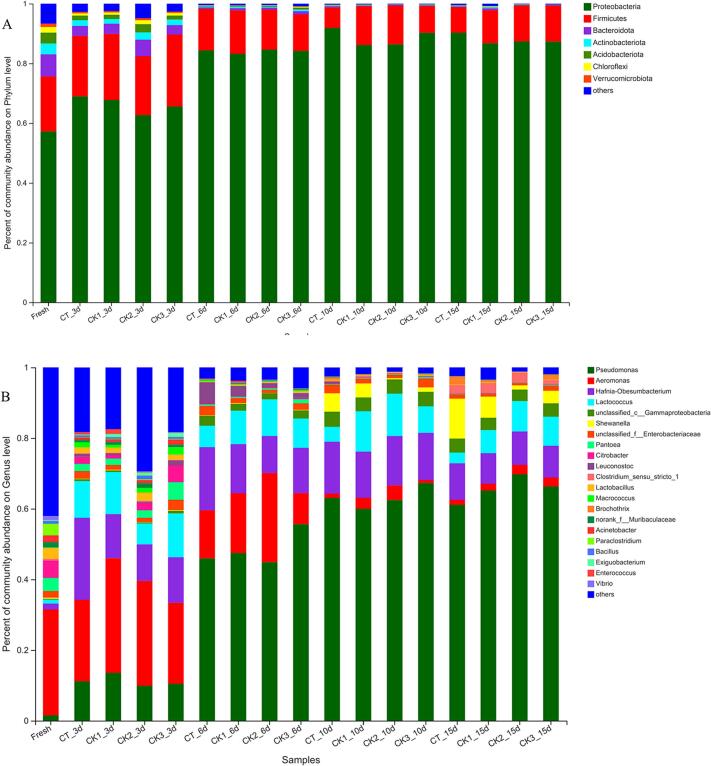
Relative abundance of bacteria at the phylum level (A) and the genus level (B) in the samples. CT: samples without glutathione peroxidase; CK1: samples with 0.1% glutathione peroxidase; CK3: samples with 0.3% glutathione peroxidase.

The relative abundance of different microorganisms at the phylum level in CMSS was shown in Fig. 2A. All samples contained similar bacterial species, but there were substantial differences in abundance. The predominant phyla in crayfish muscle were Proteobacteria and Firmicutes. As refrigeration progressed, the relative abundance of Proteobacteria in CMSS initially increased, then decreased. The rate of increase in the Proteobacteria ratio was most rapid in the CT group, rising from 57.38% to 91.84% within 10 days. Until the end of storage, Proteobacteria in the CT group were continuously higher than those in the other two groups, enunciating that GSH-Px inhibited the growth of Proteobacteria in crayfish muscle. Proteobacteria contain common spoilage bacteria in aquatic products, such as *Shewanella* and *Pseudomonas*, which play important roles in determining the quality of crayfish muscles (Liao, Wu, Li, Zhang, & Chen, 2023). Additionally, the relative abundance of Firmicutes in groups of CT and CK3 initially raised and subsequently decreased, and then tended to increase, while it exhibited a pattern of amplification followed by diminution in the CK1 group. Bacteroidota (7.37%), Actinobacteriota (3.62%), Acidobacteriota (3.61%), Chloroflexi (1.89%), Verrucomicrobiota (1.11%) and other phyla were also detected in fresh samples. There was a constant minification for the species and relative abundances of the above bacterial phyla during refrigeration. On day 15, the relative abundances of Proteobacteria and Firmicutes in the CK3 group reached 99.4%, showing the strongest dominance.

A more detailed analysis of bacterial dynamics related to crayfish refrigeration was further carried out. The top 20 genera detected in CMSS during refrigerated storage were listed in Fig. 2B. The main bacterial genera in fresh samples were *Aeromonas* (29.97%), *Citrobacter* (4.90%), *Paraclostridium* (3.26%), *Pantoea* (3.73%), *Lactobacillus* (3.10%), *Acinetobacter* (1.95%), *Enterobacteriaceae* (1.87%), *Hafnia-Obesumbacterium* (1.67%) and *Pseudomonas* (1.55%). After 3 days, macromolecular substances such as proteins in the simulated system began to degrade due to the action of endogenous and exogenous enzymes, providing nutrients for bacterial growth and reproduction. The main bacteria in CMSS changed to *Aeromonas*, *Hafnia-Obesumbacterium*, *Lactococcus*, and *Pseudomonas.* Meanwhile, it was observed that the relative abundances of *Hafnia-Obesumbacterium*, *Aeromonas,* and *Pseudomonas* in CK3 group were remarkably lower than those in the other two groups, and the relative abundance of *Hafnia-Obesumbacterium* in CK1 group was lower than that in the CT group. Therefore, GSH-Px mainly had an inhibitory effect on *Hafnia-Obesumbacterium* in the early stage of refrigeration, and the inhibitory effect was more obvious with the enzyme concentration increase. Subsequently, *Pseudomonas* began to proliferate and became the dominant genus at low temperatures, while *Hafnia-Obesumbacterium*, *Aeromonas* and *Lactococcus* gradually decreased. The relative abundances of *Shewanella*, *Gamma proteobacteria*, *Clostridium*, and *Brochothrix* were significantly increased in CMSS until the later stage of refrigeration (10 d, 15 d), especially *Shewanella*, which finally accumulated to 11.30%, 6.01% and 3.55% in groups of CT, CK1, and CK3, respectively. This indicates that *Shewanella* was inhibited by the high concentration of GSH-PX (*P* < 0.05). At the end of storage, a significant decrease in the relative abundance of *Hafnia-Obesumbacterium* and Enterobacteriaceae was also observed. After refrigerating for 15 days, the dominant spoilage bacteria in CMSS were *Pseudomonas* (61.13%), *Shewanella* (11.33%), *Hafnia-Obesumbacterium* (10.34%), *Gamma proteobacteria* (3.93%), *Lactococcus* (3.04%), *Clostridium* (2.39%), *Brochothrix* (2.21%), *Aeromonas* (1.41%), Enterobacteriaceae (1.22%), *Lactobacillus* (0.15%), among others. However, *Hafnia-Obesumbacterium* and *Shewanella* were inhibited after GSH-Px treatment (*P* < 0.05).

### 3.6 Comparison of bacterial community composition in CMSS

**Fig. 3:**
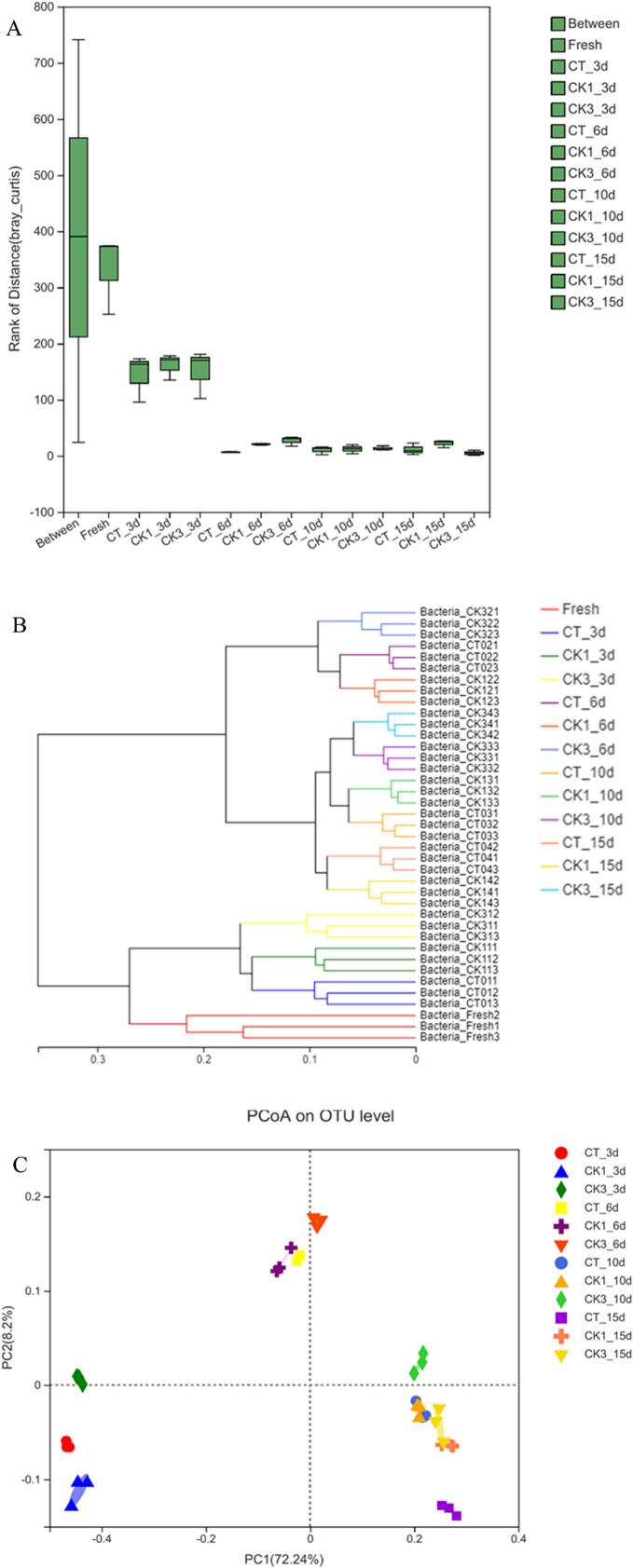
Distances box-plot between groups (A), hierarchical cluster analysis (B), and principal coordinate analysis (C) of all samples. CT: samples without glutathione peroxidase; CK1: samples with 0.1% glutathione peroxidase; CK3: samples with 0.3% glutathione peroxidase.

In order to evaluate the sample grouping of this experimental design, the distance value was calculated according to the OTU level of each sample group and a box plot of the difference between groups was drawn (Fig. 3A). The minimum, lower quartile, median, upper quartile and maximum values of the box corresponding to “Between” were 24, 212, 390.5, 566 and 741, respectively. The extremely large distance value coverage indicated that the data distribution of all samples was not concentrated, and the bacterial community structure was very different. Fresh had the largest box mean, followed by CK3_3d, CK1_3d and CT_3d, and then the remaining groups. However, the boxes in each group were smaller than those in “Between”, indicating a more concentrated data distribution within the group. Combined with the results of ANOSIM analysis (*R* = 0.8554, *P* = 0.001), the difference between the experimental groups was significantly greater than the difference within the group, suggesting good parallelism.

A hierarchical cluster analysis for all samples was presented in Fig. 3B. The community structure in CMSS on the 3rd day was considerably similar to that of the fresh samples. The three groups of samples on the 6th day were also clustered into one group, similar to the clustering observed on the 3rd day, indicating that the inhibitory effect of GSH-Px on microorganisms during the pre- and mid-refrigeration periods was weaker than that of hypothermia. In the later period of refrigeration, the microflora structure of CMSS on the 10th had a strong resemblance to that on the 15th day. In addition, the bacterial community structure exhibited similarities between CK1 group and CT group from day 1 to day 15, but differed from the CK3 group, demonstrating that high concentrations of GSH-Px had a more pronounced effect on microorganisms during storage. Particularly on the 15th day, CK3_15d was clustered with CK3_10d, CK1_10d, and CT_10d, indicating that GSH-Px exerted a strong antibacterial effect.

PCoA analysis, a principal coordinate analysis based on a distance matrix calculated from species composition, was also conducted to demonstrate microbiota differences between the two groups. As shown in Fig. 3C, the superposition of PC1 (72.24%) and PC2 (8.2%) is 80.44%, showing a good area separation. Similar to the results of the sample hierarchical clustering analysis, the points corresponding to CT_10d, CK1_10d and CK3_15d clustered together. However, there was a larger distance in PC2 between CK3_15d and CK1_15d and CK_15d, which was caused by the antibacterial properties of GSH-Px.

### 3.7 Identification of bacterial biomarkers in CMSS

**Fig. 4:**
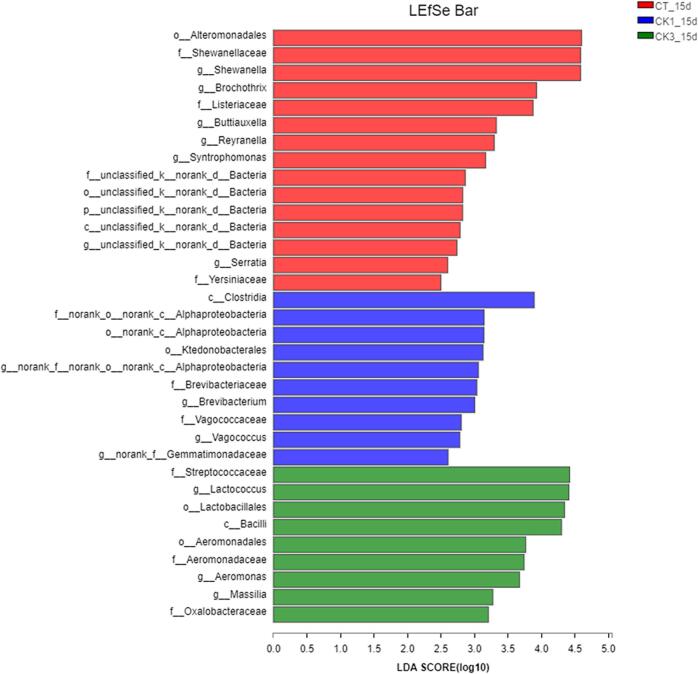
The critical species analysis of samples on day 15. CT: samples without glutathione peroxidase; CK1: samples with 0.1% glutathione peroxidase; CK3: samples with 0.3% glutathione peroxidase.

LDA discrimination can assess differences in the microbial composition between groups, and identify the microbial species with significant effect across groups. As shown in Fig. 4, the red, blue, and green columns indicate species with significantly higher abundance in the CT_15d, CK1_15d, and CK3_15d groups, respectively. The higher LDA value indicates a greater difference in effect. At the family level of microbiology, there were 9 species with significant differences among the three groups: Shewanellaceae, Listeriaceae, and Yersiniaceae in CT_15d; Alphaproteobacteria, Brevibacteriaceae and Vagococcaceae in CK1_15d; Streptococcaceae, Aeromonadaceae and Oxalobacteriaceae in CK3_15d. At the genus level, 13 genera with significant differences were identified among the three groups of samples: *Shewanella*, *Brochothrix*, *Buttiauxella*, *Reyranella*, *Syntrophomonas* and *Serratia* in the CT_15d group; *Alphaproteobacteria*, *Brevibacterium*, *Vagococcus* and *Gemmatimonadaceae* in CK1_15d group; *Lactococcus*, *Aeromonas* and *Massilia* in CK3_15d group. The differences in the genus level community structure between CK3_15d and the other two groups were mainly attributed to these genera. The above results proved that GSH-Px could significantly affect the colony structure of crayfish during storage.

### 3.8 Microbial function prediction

KEGG is a comprehensive database for systematically analyzing gene function and linking genomic information with functional information. A total of 46 metabolic pathways were identified at the L2 level of KEGG, and the top 15 metabolic pathways were shown in Table 2. Carbohydrate metabolism, amino acid metabolism, membrane transport, cellular community-prokaryotes, and xenobiotic biodegradation and metabolism exhibited an increasing trend in the GSH-Px-treated group. Among these pathways, carbohydrate metabolism had the highest relative abundance, reaching approximately 8.35%. Carbohydrate metabolism encompassed several L3 metabolic pathways, including pyruvate metabolism, glyoxylate, and dicarboxylate metabolism, glycolysis/gluconeogenesis, amino sugar and nucleotide sugar metabolism, starch and sucrose metabolism, pentose phosphate pathway, citrate cycle (TCA cycle), and fructose and mannose metabolism. These pathways provide essential nutrients for bacterial growth and reproduction (Georges, 2016). Additionally, the high concentration of carbon dioxide in the environment, produced by glycolysis and the tricarboxylic acid cycle, was conducive to the growth and reproduction of facultative anaerobic bacteria such as *Lactococcus* and *Aeromonas*. These bacteria ferment carbohydrates and produce acidic substances, which may explain the lower pH in the CK3_15d group. Moreover, membrane transport was also significantly affected by GSH-Px. The relative abundances of the phosphotransferase system (from 0.32% to 0.36%) and ABC transporters (from 3.22% to 3.40%) in its L3 metabolic pathway (Table S1) were up-regulated by 0.3% GSH-Px, while bacterial secretion systems changed less. The phosphotransferase system is the main mechanism for bacteria to absorb carbohydrates (Deutscher, Francke, & Postma, 2006). ABC transporters widely present in bacteria, archaea and eukaryotes mainly combine ATP hydrolysis with active transport of various substrates (https://www.kegg.jp/kegg/pathway.html), thus membrane transport rise in GSH-Px-treated group was probably closely related to the increase of *Lactococcus* and *Aeromonas*. GSH-Px might also upregulate quorum sensing and biofilm formation in *Pseudomonas aeruginosa*, while affected obviously on biofilm formation of *Vibrio cholerae* and *Escherichia coli*. Additionally, the metabolism of cofactors and vitamins, energy metabolism, signal transduction, metabolism of other amino acids, and cell motility all showed a downward trend with the addition of GSH-Px. Nucleotide metabolism, translation, lipid metabolism, and replication and repair metabolic pathways showed less impact from GSH-Px.

**Table 2:** KEGG function prediction (L2 level) at 15 days of refrigerated storage. CT: samples without glutathione peroxidase; CK1: samples with 0.1% glutathione peroxidase; CK3: samples with 0.3% glutathione peroxidase.

KEGG_L2 metabolic pathways	CT_15d	CK1_15d	CK3_15d
Global and overview maps	37.61%	37.56%	37.64%
Carbohydrate metabolism	8.30%	8.30%	8.46%
Amino acid metabolism	7.81%	7.86%	7.85%
Membrane transport	4.64%	4.76%	4.86%
Metabolism of cofactors and vitamins	4.25%	4.20%	4.13%
Energy metabolism	4.11%	4.08%	4.03%
Signal transduction	4.27%	4.28%	4.21%
Cellular community - prokaryotes	3.21%	3.24%	3.23%
Nucleotide metabolism	2.25%	2.25%	2.24%
Translation	2.06%	2.07%	2.09%
Lipid metabolism	2.17%	2.18%	2.17%
Replication and repair	2.09%	2.10%	2.09%
Xenobiotics biodegradation and metabolism	2.03%	2.03%	2.08%
Metabolism of other amino acids	1.64%	1.61%	1.62%
Cell motility	1.65%	1.58%	1.49%

## 4 Conclusions

During refrigerated storage (3.0 ± 1.0 °C), GSH-Px treatment was more beneficial to maintain the high quality of crayfish without excessive accumulation of TVB-N and TVC. The CT and CK3 groups exhibited significant differences in the relative abundance of spoilage microorganisms, such as *Hafnia-Obesumbacterium* and *Shewanella* in Proteobacteria. The CK3_15d, CK1_15d, and CT groups demonstrated differential expression in the genera *Lactococcus*, *Aeromonas*, and *Massilia*. The colony composition of the CK3_15d and CT_10d groups was observed to be aggregated together. The results indicate that GSH-Px inhibited the degradation of nitrogenous substance (*P* < 0.05), thereby prolonging the shelf life of crayfish through the suppression of spoilage microorganism proliferation. This study could provide a theoretical foundation for the potential application of a novel enzyme-based biological preservative for crayfish.

## CRediT authorship contribution statement

**Lerong Qin:** Writing – original draft, Formal analysis, Data curation. **Han Li:** Software, Data curation, Visualization. **Ying Zhang:** Conceptualization, Methodology. **Jiwang Chen:** Methodology, Resources. **Haibin Wang:** Methodology, Resources. **E. Liao:** Writing – review & editing, Writing – original draft, Conceptualization, Funding acquisition, Methodology, Project administration.

## Declaration of competing interest

The authors attested that there were no conflicts of interest with respect to this paper.

## Data Availability

Data will be made available on request.

## Appendix A Supplementary data

Supplementary material Image 1

## References

[bb0005] Adebayo-Tayo B.C., Yusuf B.O., Alao S.O. (2021). Antibacterial activity of intracellular greenly fabricated selenium nanoparticle of *Lactobacillus pentosus* ADET MW861694 against selected food pathogens. The International Journal of Biotechnology.

[bb0010] Balti R., Ben Mansour M., Zayoud N., Le Balc'h R., Brodu N., Arhaliass A., Massé A. (2020). Active exopolysaccharides based edible coatings enriched with red seaweed (*Gracilaria gracilis*) extract to improve shrimp preservation during refrigerated storage. Food Bioscience.

[bb0015] Brigelius-Flohé R., Flohé L. (2020). Regulatory phenomena in the glutathione peroxidase superfamily. Antioxidants & Redox Signaling.

[bb0020] Cermelli C., Vinceti M., Scaltriti E., Bazzani E., Beretti F., Vivoli G., Portolani M. (2002). Selenite inhibition of Coxsackie virus B5 replication: Implications on the etiology of Keshan disease. Journal of Trace Elements in Medicine and Biology.

[bb0025] Deutscher J., Francke C., Postma P. (2006). How phosphotransferase system-related protein phosphorylation regulates carbohydrate metabolism in bacteria. Microbiology and Molecular Biology Reviews.

[bb0030] Edgar R.C. (2013). UPARSE: Highly accurate OTU sequences from microbial amplicon reads. Nature Methods.

[bb0035] Edgar R.C., Haas B.J., Clemente J.C., Quince C., Knight R. (2011). UCHIME improves sensitivity and speed of chimera detection. Bioinformatics.

[bb0040] Ge Y., Li Y., Wu T., Bai Y., Yuan C., Chen S., Hu Y. (2020). The preservation effect of CGA-gel combined with partial freezing on sword prawn (*Parapenaeopsis hardwickii*). Food Chemistry.

[bb0045] Georges N. (2016).

[bb0050] Gram L., Dalgaard P. (2002). Fish spoilage bacteria-problems and solutions. Current Opinion in Biotechnology.

[bb0055] Han H.W., Patel K.D., Kwak J.H., Jun S.K., Jang T.S., Lee S.H., Lee J.H. (2021). Selenium nanoparticles as candidates for antibacterial substitutes and supplements against multidrug-resistant bacteria. Biomolecules.

[bb0060] Lankin V.Z., Shumaev K.B., Tikhaze A.K., Kurganov B.I. (2017). Influence of dicarbonyls on kinetic characteristics of glutathione peroxidase. Doklady Biochemistry and Biophysics.

[bb0065] Li S., Zhao Q., Zhang K., Sun W., Jia X., Yang Y., Yin J., Tang C., Zhang J. (2020). Se deficiency induces renal pathological changes by regulating selenoprotein expression, disrupting redox balance, and activating inflammation. Metallomics.

[bb0070] Liao E., Wu Y., Li Q., Zhang Y., Chen J. (2023). Isolation and identification of specific spoilage organisms and analysis of saprogenic amine production capacity during micro-frozen storage of *Procambarus clarkii*. Journal of Food Safety and Quality.

[bb0075] Liao E., Wu Y., Pan Y., Zhang Y., Zhang P., Chen J. (2023). Cryoprotective effects of carrageenan oligosaccharides on crayfish (*Procambarus clarkii*) during superchilling. Foods.

[bb0080] López-Caballero M.E., Martínez-Alvarez O., Gómez-Guillén M.D.C., Montero P. (2007). Quality of thawed Deepwater pink shrimp (*Parapenaeus longirostris*) treated with melanosis-inhibiting formulations during chilled storage. International Journal of Food Science & Technology.

[bb0085] Mei J., Ma X., Xie J. (2019). Review on natural preservatives for extending fish shelf life. Foods.

[bb0090] Na S., Kim J.H., Jang H.J., Park H.J., Oh S.W. (2018). Shelf life extension of Pacific white shrimp (*Litopenaeus vannamei*) using chitosan and ε-polylysine during cold storage. International Journal of Biological Macromolecules.

[bb0100] Peng S., Wei H., Zhan S., Yang W., Luo Q., Deng S., Yu X., Huang T. (2022). Spoilage mechanism and preservation technologies on the quality of shrimp: An overview. Trends in Food Science & Technology.

[bb0105] Qin L., Wu Y., Chen J., Xia W., Liao E., Wang H. (2022). Effects of superchilling on quality of crayfish (*Procambarus clarkii*): Water migration, biogenic amines accumulation, and nucleotides catabolism. International Journal of Food Science & Technology.

[bb0110] dos Santos Souza L.M., Dibo M., Sarmiento J.J.P., Seabra A.B., Medeiros L.P., Lourenço I.M., Nakazato G. (2022). Biosynthesis of selenium nanoparticles using combinations of plant extracts and their antibacterial activity. Current Research in Green and Sustainable Chemistry.

[bb0115] Shokri S., Ehsani A. (2017). Efficacy of whey protein coating incorporated with lactoperoxidase and alpha-tocopherol in shelf life extension of pike-perch fillets during refrigeration. LWT - Food Science and Technology.

[bb0120] Stefanello S.T., Mizdal C.R., Gonçalves D.F., Hartmann D.D., Dobrachinski F., de Carvalho N.R., Soares F.A.A. (2020). The insertion of functional groups in organic selenium compounds promote changes in mitochondrial parameters and raise the antibacterial activity. Bioorganic Chemistry.

[bb0125] Wan J., Zhang M., Adhikari B. (2018). Advances in selenium-enriched foods: From the farm to the fork. Trends in Food Science & Technology.

[bb0130] Wang H., Chen X., Zhang J., Wang X., Shi W. (2020). Postmortem changes in the freshness and volatile compounds of grass carp (*Ctenopharyngodon idella*). Journal of Food Measurement and Characterization.

[bb0135] Wang Z., Hu S., Gao Y., Wang H. (2017). Effect of collagen-lysozyme coating on fresh-salmon fillets preservation. LWT - Food Science and Technology.

[bb0140] Wu M., Zhu Z., Li S., Cai J., Cong X., Yu T., Cheng S. (2020). Green recovery of se-rich protein and antioxidant peptides from Cardamine Violifolia: Composition and bioactivity. Food Bioscience.

[bb0145] Yuan M., Ning C., Yang S., Liang Q., Mou H., Liu Z. (2020). A new cold-active glucose oxidase from Penicillium: High-level expression and application in fish preservation. Frontiers in Microbiology.

[bb0150] Zang J., Xu Y., Xia W., Yu D., Gao P., Jiang Q., Yang F. (2018). Dynamics and diversity of microbial community succession during fermentation of Suan yu, a Chinese traditional fermented fish, determined by high throughput sequencing. Food Research International.

[bb0155] Zhang B., Mao J.L., Yao H., Aubourg S.P. (2020). Label-free based proteomics analysis of protein changes in frozen whiteleg shrimp (*Litopenaeus vannamei*) pre-soaked with sodium trimetaphosphate. Food Research International.

[bb0160] Zhang B., Zhao J., Chen S., Zhang X., Wei W. (2019). Influence of trehalose and alginate oligosaccharides on ice crystal growth and recrystallization in whiteleg shrimp (*Litopenaeus vannamei*) during frozen storage with temperature fluctuations. International Journal of Refrigeration.

[bb0165] Zhang K., Zhao Q., Zhan T., Han Y., Tang C., Zhang J. (2020). Effect of different selenium sources on growth performance, tissue selenium content, meat quality, and selenoprotein gene expression in finishing pigs. Biological Trace Element Research.

[bb0170] Zhou F., Yang W., Wang M., Miao Y., Cui Z., Li Z., Liang D. (2018). Effects of selenium application on se content and speciation in Lentinula edodes. Food Chemistry.

[bb0175] Zwolak I. (2020). The role of selenium in arsenic and cadmium toxicity: An updated review of scientific literature. Biological Trace Element Research.

