# Supplementary material for: Inhibitory effects of glutathione peroxidase on microbial spoilage of crayfish (Procambarus clarkii) during refrigerated storage
Source: Food Chem X. 2024 Apr 13;22:101388. doi: 10.1016/j.fochx.2024.101388 (PMC11043841; doi:10.1016/j.fochx.2024.101388)
Supplement: Supplementary file 1 — Supplementary material [file mmc1.docx]

Table S1 KEGG function prediction (L3 level) at 0, 3, 6, 10, and 15 days of refrigerated storage. CT: samples without glutathione peroxidase; CK1: samples with 0.1% glutathione peroxidase; CK3: samples with 0.3% glutathione peroxidase.

| KEGG_L3 metabolic pathways | CK1_10d | CK1_15d | CK1_3d | CK1_6d | CK3_10d | CK3_15d | CK3_3d | CK3_6d | CT_10d | CT_15d | CT_3d | CT_6d | Fresh |
| --- | --- | --- | --- | --- | --- | --- | --- | --- | --- | --- | --- | --- | --- |
| Metabolic pathways | 17.82% | 17.81% | 17.93% | 17.80% | 17.81% | 17.78% | 18.07% | 17.80% | 17.82% | 17.81% | 18.04% | 17.82% | 18.15% |
| Biosynthesis of secondary metabolites | 7.72% | 7.70% | 8.13% | 7.68% | 7.63% | 7.71% | 8.13% | 7.65% | 7.63% | 7.72% | 8.09% | 7.64% | 8.20% |
| Microbial metabolism in diverse environments | 4.90% | 4.89% | 4.89% | 4.94% | 4.96% | 4.91% | 4.95% | 4.95% | 4.96% | 4.91% | 4.99% | 4.97% | 4.96% |
| Two-component system | 3.54% | 3.63% | 2.82% | 3.53% | 3.61% | 3.56% | 2.80% | 3.57% | 3.65% | 3.62% | 2.80% | 3.56% | 2.56% |
| ABC transporters | 3.38% | 3.30% | 3.04% | 3.41% | 3.48% | 3.40% | 3.23% | 3.46% | 3.39% | 3.22% | 3.07% | 3.44% | 2.78% |
| Biosynthesis of amino acids | 3.04% | 2.99% | 3.29% | 3.00% | 2.96% | 3.03% | 3.32% | 2.97% | 2.93% | 2.98% | 3.24% | 2.96% | 3.30% |
| Carbon metabolism | 2.47% | 2.45% | 2.64% | 2.47% | 2.44% | 2.45% | 2.64% | 2.46% | 2.45% | 2.47% | 2.64% | 2.46% | 2.70% |
| Purine metabolism | 1.43% | 1.43% | 1.54% | 1.42% | 1.41% | 1.42% | 1.53% | 1.40% | 1.41% | 1.42% | 1.53% | 1.42% | 1.52% |
| Quorum sensing | 1.39% | 1.36% | 1.52% | 1.43% | 1.39% | 1.38% | 1.51% | 1.41% | 1.35% | 1.32% | 1.48% | 1.42% | 1.54% |
| Ribosome | 1.33% | 1.31% | 1.62% | 1.31% | 1.27% | 1.32% | 1.61% | 1.28% | 1.26% | 1.30% | 1.63% | 1.29% | 1.71% |
| Oxidative phosphorylation | 1.07% | 1.11% | 1.04% | 1.07% | 1.08% | 1.07% | 1.01% | 1.08% | 1.10% | 1.11% | 1.04% | 1.08% | 1.09% |
| Bacterial secretion system | 1.12% | 1.15% | 0.94% | 1.17% | 1.13% | 1.11% | 0.87% | 1.16% | 1.14% | 1.10% | 0.89% | 1.16% | 0.80% |
| Pyruvate metabolism | 1.01% | 0.99% | 1.07% | 1.00% | 1.00% | 1.01% | 1.08% | 0.99% | 0.99% | 1.00% | 1.08% | 1.00% | 1.08% |
| Glyoxylate and dicarboxylate metabolism | 1.00% | 1.02% | 0.82% | 1.00% | 1.04% | 1.01% | 0.81% | 1.04% | 1.04% | 1.02% | 0.82% | 1.01% | 0.83% |
| Glycine, serine and threonine metabolism | 0.94% | 0.93% | 0.91% | 0.95% | 0.95% | 0.94% | 0.90% | 0.95% | 0.94% | 0.93% | 0.90% | 0.95% | 0.89% |
| Cysteine and methionine metabolism | 0.86% | 0.86% | 0.91% | 0.85% | 0.84% | 0.86% | 0.92% | 0.85% | 0.84% | 0.86% | 0.90% | 0.84% | 0.92% |
| Pyrimidine metabolism | 0.83% | 0.82% | 1.00% | 0.83% | 0.80% | 0.82% | 1.01% | 0.80% | 0.79% | 0.82% | 1.00% | 0.82% | 1.03% |
| Glycolysis / Gluconeogenesis | 0.80% | 0.75% | 1.02% | 0.80% | 0.77% | 0.79% | 1.05% | 0.77% | 0.75% | 0.76% | 1.03% | 0.79% | 1.02% |
| Amino sugar and nucleotide sugar metabolism | 0.76% | 0.73% | 1.00% | 0.77% | 0.73% | 0.75% | 1.02% | 0.74% | 0.72% | 0.73% | 0.99% | 0.76% | 1.04% |
| Flagellar assembly | 0.78% | 0.83% | 0.84% | 0.80% | 0.75% | 0.78% | 0.76% | 0.76% | 0.82% | 0.90% | 0.78% | 0.80% | 0.85% |
| Propanoate metabolism | 0.79% | 0.81% | 0.75% | 0.80% | 0.81% | 0.80% | 0.74% | 0.81% | 0.81% | 0.81% | 0.76% | 0.81% | 0.74% |
| Fatty acid metabolism | 0.79% | 0.80% | 0.71% | 0.79% | 0.81% | 0.80% | 0.68% | 0.81% | 0.81% | 0.79% | 0.70% | 0.79% | 0.69% |
| Porphyrin and chlorophyll metabolism | 0.78% | 0.83% | 0.62% | 0.77% | 0.80% | 0.79% | 0.62% | 0.79% | 0.82% | 0.83% | 0.64% | 0.78% | 0.68% |
| Carbon fixation pathways in prokaryotes | 0.74% | 0.75% | 0.77% | 0.73% | 0.73% | 0.74% | 0.76% | 0.73% | 0.74% | 0.76% | 0.77% | 0.73% | 0.84% |
| Alanine, aspartate and glutamate metabolism | 0.72% | 0.72% | 0.76% | 0.71% | 0.71% | 0.71% | 0.75% | 0.71% | 0.71% | 0.73% | 0.75% | 0.71% | 0.79% |
| Butanoate metabolism | 0.70% | 0.71% | 0.70% | 0.70% | 0.71% | 0.71% | 0.69% | 0.70% | 0.72% | 0.73% | 0.71% | 0.71% | 0.74% |
| Bacterial chemotaxis | 0.70% | 0.74% | 0.64% | 0.72% | 0.70% | 0.71% | 0.60% | 0.71% | 0.73% | 0.76% | 0.60% | 0.72% | 0.64% |
| Aminoacyl-tRNA biosynthesis | 0.67% | 0.65% | 0.80% | 0.66% | 0.64% | 0.66% | 0.80% | 0.64% | 0.63% | 0.64% | 0.79% | 0.64% | 0.84% |
| Starch and sucrose metabolism | 0.66% | 0.60% | 0.87% | 0.64% | 0.62% | 0.65% | 0.93% | 0.60% | 0.58% | 0.60% | 0.88% | 0.63% | 0.79% |
| Sulfur metabolism | 0.69% | 0.71% | 0.54% | 0.69% | 0.72% | 0.71% | 0.57% | 0.71% | 0.73% | 0.72% | 0.55% | 0.70% | 0.54% |
| 2-Oxocarboxylic acid metabolism | 0.67% | 0.66% | 0.69% | 0.66% | 0.65% | 0.66% | 0.69% | 0.65% | 0.65% | 0.66% | 0.68% | 0.65% | 0.70% |
| Pentose phosphate pathway | 0.66% | 0.63% | 0.68% | 0.64% | 0.65% | 0.65% | 0.73% | 0.64% | 0.64% | 0.63% | 0.72% | 0.64% | 0.66% |
| Valine, leucine and isoleucine degradation | 0.68% | 0.72% | 0.51% | 0.69% | 0.72% | 0.70% | 0.45% | 0.72% | 0.72% | 0.71% | 0.49% | 0.69% | 0.47% |
| Biofilm formation - Pseudomonas aeruginosa | 0.68% | 0.70% | 0.45% | 0.71% | 0.72% | 0.68% | 0.43% | 0.74% | 0.71% | 0.67% | 0.44% | 0.72% | 0.33% |
| Citrate cycle (TCA cycle) | 0.63% | 0.62% | 0.64% | 0.62% | 0.62% | 0.62% | 0.63% | 0.62% | 0.62% | 0.63% | 0.63% | 0.62% | 0.65% |
| Homologous recombination | 0.62% | 0.61% | 0.68% | 0.60% | 0.60% | 0.61% | 0.68% | 0.59% | 0.60% | 0.62% | 0.68% | 0.59% | 0.69% |
| Folate biosynthesis | 0.62% | 0.64% | 0.59% | 0.62% | 0.62% | 0.62% | 0.57% | 0.62% | 0.64% | 0.65% | 0.58% | 0.63% | 0.58% |
| Methane metabolism | 0.56% | 0.55% | 0.69% | 0.57% | 0.55% | 0.55% | 0.70% | 0.56% | 0.56% | 0.57% | 0.70% | 0.57% | 0.73% |
| Phenylalanine, tyrosine and tryptophan biosynthesis | 0.58% | 0.57% | 0.61% | 0.57% | 0.57% | 0.58% | 0.62% | 0.57% | 0.56% | 0.57% | 0.59% | 0.56% | 0.61% |
| Peptidoglycan biosynthesis | 0.57% | 0.55% | 0.66% | 0.56% | 0.55% | 0.56% | 0.67% | 0.55% | 0.53% | 0.54% | 0.66% | 0.55% | 0.64% |
| Arginine and proline metabolism | 0.59% | 0.61% | 0.50% | 0.60% | 0.61% | 0.60% | 0.49% | 0.61% | 0.62% | 0.61% | 0.50% | 0.60% | 0.50% |
| Biofilm formation - Escherichia coli | 0.58% | 0.58% | 0.52% | 0.59% | 0.60% | 0.58% | 0.54% | 0.59% | 0.61% | 0.59% | 0.53% | 0.60% | 0.48% |
| Biofilm formation - Vibrio cholerae | 0.58% | 0.60% | 0.55% | 0.59% | 0.58% | 0.59% | 0.48% | 0.58% | 0.61% | 0.63% | 0.50% | 0.59% | 0.51% |
| Arginine biosynthesis | 0.59% | 0.60% | 0.50% | 0.59% | 0.60% | 0.59% | 0.49% | 0.60% | 0.59% | 0.57% | 0.48% | 0.58% | 0.46% |
| Mismatch repair | 0.52% | 0.52% | 0.57% | 0.51% | 0.50% | 0.52% | 0.58% | 0.50% | 0.50% | 0.52% | 0.58% | 0.50% | 0.59% |
| Fructose and mannose metabolism | 0.52% | 0.49% | 0.55% | 0.51% | 0.50% | 0.51% | 0.63% | 0.50% | 0.47% | 0.46% | 0.56% | 0.50% | 0.56% |
| Cationic antimicrobial peptide (CAMP) resistance | 0.52% | 0.52% | 0.46% | 0.52% | 0.54% | 0.53% | 0.49% | 0.52% | 0.55% | 0.54% | 0.49% | 0.53% | 0.44% |
| Lipopolysaccharide biosynthesis | 0.52% | 0.53% | 0.43% | 0.53% | 0.54% | 0.52% | 0.44% | 0.53% | 0.55% | 0.53% | 0.45% | 0.54% | 0.44% |
| Fatty acid biosynthesis | 0.50% | 0.50% | 0.52% | 0.50% | 0.49% | 0.50% | 0.50% | 0.49% | 0.49% | 0.49% | 0.51% | 0.49% | 0.51% |
| Glycerophospholipid metabolism | 0.48% | 0.48% | 0.50% | 0.48% | 0.48% | 0.48% | 0.51% | 0.48% | 0.48% | 0.48% | 0.51% | 0.49% | 0.51% |
| Glutathione metabolism | 0.49% | 0.49% | 0.41% | 0.48% | 0.49% | 0.49% | 0.41% | 0.49% | 0.50% | 0.51% | 0.42% | 0.49% | 0.39% |
| Histidine metabolism | 0.49% | 0.49% | 0.41% | 0.48% | 0.49% | 0.49% | 0.42% | 0.48% | 0.48% | 0.48% | 0.39% | 0.47% | 0.40% |
